# Prevalence, severity, frequency and healthcare resource use of epilepsy among individuals with Rett Syndrome: analysis of data from a Rett Center of Excellence

**DOI:** 10.3389/fneur.2025.1634105

**Published:** 2025-10-10

**Authors:** Nazia Rashid, Charles Ruetsch, Emily M. LaFrance, Jonathan D. Darer

**Affiliations:** ^1^Acadia Pharmaceuticals, Medical Affairs, San Diego, CA, United States; ^2^Health Analytics, LLC, Clarksville, MD, United States

**Keywords:** Rett Syndrome, epilepsy, seizures, healthcare utilization, electronic health records

## Abstract

**Background:**

Individuals with Rett Syndrome (RTT) frequently experience comorbid epilepsy. Few studies have examined the impact of epilepsy healthcare utilization using real-world datasets among RTT. The objective of this study was to examine all-cause and epilepsy-related healthcare utilization among individuals with RTT using electronic health record (EHR) data and chart notes.

**Methods:**

A retrospective comparative cohort analysis was performed among RTT individuals with and without epilepsy using EHR structured and abstracted clinical notes data. Epilepsy cases were stratified into severe (associated with prolonged or intractable seizures) and non-severe. RTT individuals with no epilepsy, non-severe epilepsy, and severe epilepsy were compared on seizure frequency and healthcare utilization.

**Results:**

In this analysis, 98 individuals with RTT were included: 71 (72%) had epilepsy of which 33 (46%) had severe epilepsy. Individuals with severe epilepsy (*n* = 33) vs. non-severe epilepsy [experienced daily seizures (53.8% vs. 11.1%), weekly (23.1% vs. 14.8%), or monthly (23.1% vs. 22.2%)], respectively; however, more than half (51.9%) experienced no seizures among the non-severe group. The prevalence of ED visits was significantly higher among those with severe epilepsy compared to those with non-severe epilepsy (27% vs. 7%, *p* < 0.05). Inpatient admissions were significantly more prevalent among those with severe epilepsy vs. non-severe epilepsy (37% vs. 14%, *p* < 0.05).

**Conclusions:**

Rates of epilepsy among individuals with RTT are at increased risk for healthcare utilization, especially those with severe epilepsy described as prolonged or intractable seizures. Of individuals with epilepsy, up to one-fourth experience daily seizures. It is important to educate health professionals of the high rate of concomitant epilepsy among RTT syndrome and how it can represent a substantial burden to patients and families with RTT. The frequency of seizures is more common among those with severe epilepsy.

## 1 Introduction

Rett syndrome (RTT) is a rare neurodevelopmental disorder predominantly affecting females (1, 2). Prevalence of RTT is estimated at 7.1 per 100,000 females (2) and is a leading cause of intellectual disability in young girls (3). Individuals with RTT characteristically have normal neuropsychic development for the first 6–18 months of life and then experience a progressive deterioration of acquired behavioral and motor skills (4). RTT impacts multiple physiological domains including growth, development, gastrointestinal, respiratory, musculoskeletal, and neurologic function (5, 6). The diagnosis of RTT is made clinically, though there are strong associations with mutations in the methyl-CpG-binding protein 2 gene [MECP2; (7)].

A common neurologic comorbidity associated with RTT syndrome is epilepsy, affecting 50% to 90% of individuals with RTT (7, 8). Seizures tend to present between the ages of 3 and 20 (9) with peak frequency reported in ages 7–12 (10). Incidence of epilepsy is higher in individuals with RTT who experience more severe developmental disabilities (11). Seizure types in RTT vary and include focal onset, complex partial, atypical absence, atonic, and generalized tonic-myoclonic (9, 12). Generalized seizures are correlated with treatment-resistance while seizure presentation after age 5 predicts future control (13). Genetic variants of RTT can be associated with different epilepsy presentation. Individuals with cyclin-dependent kinase-like 5 (CDKL5) variants generally present with epilepsy before age 1 while individuals with Forkhead Box G1 (FOXG1) variants generally present with epilepsy at 2 years of age with drug resistant seizures (13). The association of genetic variants of MECP2 mutations with epilepsy ranges from an estimated 48%-100% (12). Only 20% of individuals with epilepsy in RTT achieve seizure control (9).

Families and caregivers are negatively burdened by epilepsy, especially among those who experience generalized, prolonged, cyanotic, or refractory seizures (14, 15). Parents of individuals with RTT and epilepsy can experience fear of seizures as well as difficulty in finding home care for their children (14).

Real-world evidence regarding epilepsy in RTT, frequency, severity of seizures, and their impact upon healthcare resource utilization (HCRU) is limited. Claims-based analysis frequently underreports symptoms and clinical events using diagnosis codes only. Thus, having access to clinical chart progress notes can provide important information regarding seizure frequency.

Currently, there is limited published real-world data providing detailed information about seizures among RTT syndrome. To better understand the prevalence, seizure severity (severe vs. non-severe), frequency (daily, weekly, monthly), and HCRU related to epilepsy among individuals with RTT syndrome, we completed a retrospective database study with structured (diagnosis codes) and unstructured data (clinical progress notes) among individuals with RTT receiving care at a RTT Center of Excellence in the United States.

## 2 Methods

### 2.1 Data source

Data for these analyses were licensed from Nashville Biosciences (www.nashville.bio) and included electronic health record (EHR) data from Vanderbilt University Medical Center (VUMC) including diagnoses codes based on International Classification of Diseases, 9th and 10th Revision, Clinical Modification (ICD-9-CM, ICD-10-CM) codes (Supplementary Table 1), outpatient encounters (dates of service, provider specialty type from visit, encounter diagnoses), inpatient admissions (hospitalizations), emergency department (ED) visits, and orders (medications, laboratory tests, procedures, and imaging exams). Data was also abstracted from clinical progress notes using the notes variable list which were then linked to structured data at the patient level by Nashville Biosciences prior to de-identification. In addition to diagnosis codes, data regarding epilepsy symptoms, and severity of seizures were abstracted from clinical progress notes assessing the initial epilepsy evaluation by a neurologist. The clinical progress notes were reviewed by a team of clinicians experienced with the dataset and identified the epilepsy cases. Additionally, frequency of seizures was abstracted from neurologist clinical progress notes post-index epilepsy diagnosis only. The VUMC is recognized as one of the Rett Center of Excellence facilities where they provide coordinated multidisciplinary care for children with RTT syndrome and RTT-related disorders. Data were de-identified and compliant with the Health Insurance Portability and Accountability Act.

### 2.2 Study design

A retrospective, cohort study design was used to complete this chart and database analysis. Eligible study participants were identified and selected from individuals with RTT receiving care from the VUMC during 02/14/1990 to 08/31/2023. The index date was defined as the date of the first epilepsy event (case) observed by: a diagnosis code from a medical encounter, or abstracted from a clinical progress note, or medication to treat epilepsy. Prior to index date was labeled as baseline/pre-index period and on index date to 12 months after index date was labeled as post-index period.

### 2.3 Patient population

**Figure 1:**
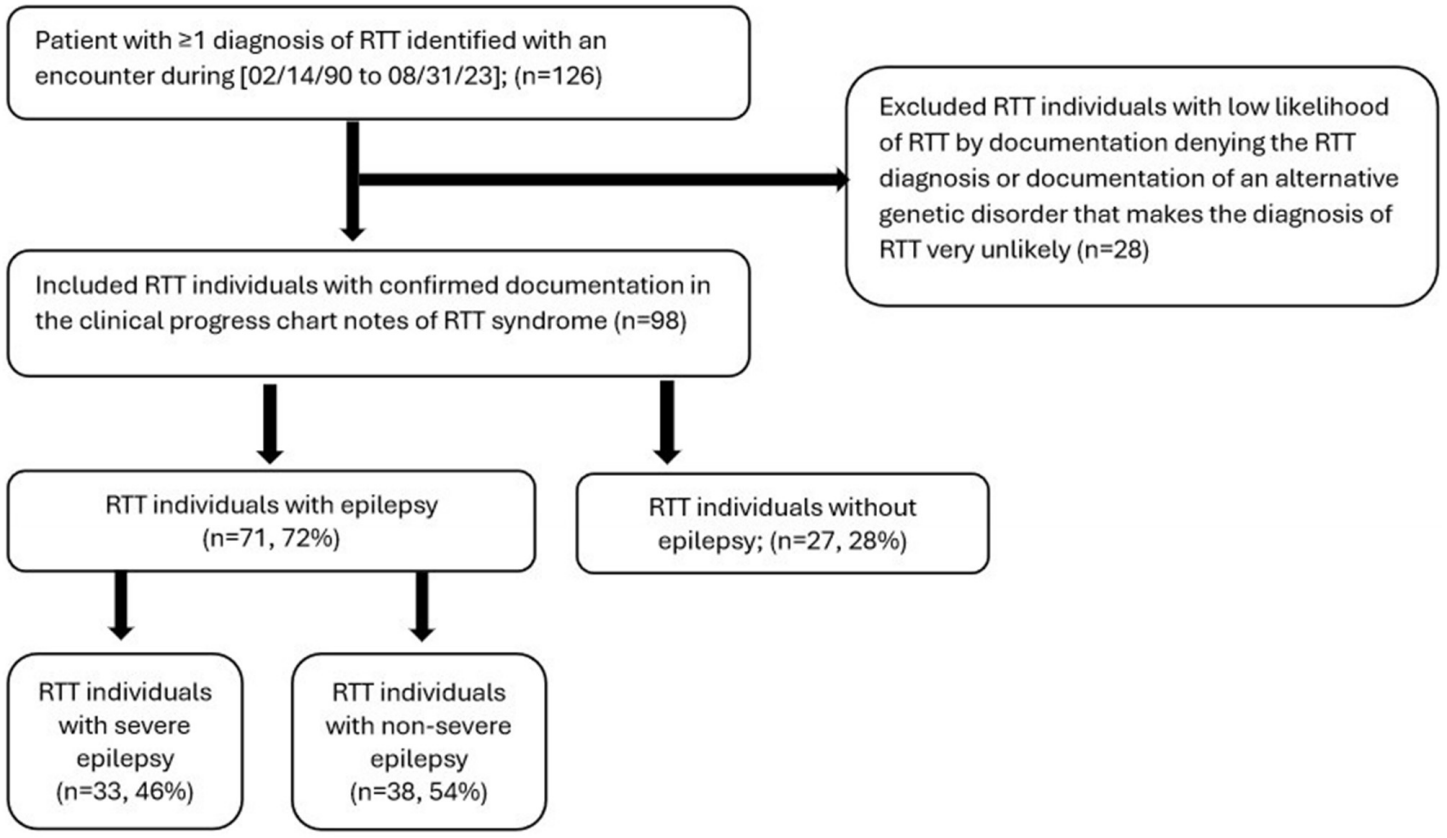
Attrition diagram of study groups.

To be eligible for inclusion in this study, individuals were required to have the following criteria during 2/14/1990 to 8/31/2023: (a) ≥1 medical encounter (outpatient, inpatient, or emergency department) with a coded diagnosis of RTT (ICD-10 F82.4), (b) a clinical progress note confirming the presence of RTT. RTT Individuals with a clinical progress note denying the presence of RTT were excluded from the study (Figure 1).

To evaluate prevalence, all patients included in the study with RTT syndrome were assessed with epilepsy vs. no-epilepsy. Epilepsy cases were determined by (a) the presence of an ICD-9 (345.xx) or ICD-10 (G40.xx) code for epilepsy associated with an outpatient visit, inpatient admission, or ED visit in any position, (b) the presence of clinician-confirmed epilepsy in a clinical progress note, or (c) the presence of an anti-epilepsy drug (AED). The epilepsy cohort was further categorized into severe epilepsy and non-severe epilepsy. Individuals with severe epilepsy were identified as those with (a) either an ICD-10 code indicating status epilepticus (G40.XX1), or intractable epilepsy (G40.3X, G40A1X, G40.B1X, G40.C1X, G40.41X, G40.8X3, G40.8X4, G40.91X) per Supplementary Table 1, or (b) clinical progress notes documenting prolonged, cyanotic, or refractory seizures. In the absence of criteria for severe epilepsy, individuals were assumed to have non-severe epilepsy. Frequency of seizures was assessed and abstracted from neurologist clinical progress notes post-index epilepsy diagnosis. To assess HCRU, three different analyses were completed: (a) evaluating all-cause HCRU among RTT individuals with epilepsy and their severity during 12 months post-index; (b) evaluating epilepsy-specific HCRU among RTT individuals with epilepsy and their severity during 12 months post-index; (c) evaluating all-cause HCRU among RTT individuals with epilepsy who have 12 months pre-index data and 12 months post-index data (pre- and post-analysis).

### 2.4 Study outcomes

Among the RTT individuals included in the study, prevalence of epilepsy was identified using diagnosis codes, progress clinical notes and medications. Epilepsy was then categorized by severity (severe vs. non-severe) using diagnosis codes and clinical progress notes; frequency of seizures was only available for those that had frequency (daily, weekly or monthly) information from their neurologist epilepsy evaluations post-index documented in clinical progress notes. AED utilization among epilepsy cases was also determined during the 12 months post-index. HCRU (outpatient visits, ED visits, and inpatient admissions) was assessed by all-cause and epilepsy-specific (primary or secondary code of claims for diagnosis of epilepsy) during the 12 months post-index; HCRU was also assessed for those that had 12 months pre-index data and 12 months post-index data (pre/post-index analysis).

### 2.5 Statistical analysis

Patient demographics, clinical characteristics, and outcome measures were reported descriptively as frequencies and percentages for categorical variables; mean, standard deviation (SD) for were reported for continuous variables. A *p*-value of < 0.05 was defined as statistical significance. Prevalence of HCRU (number and percentage of patients with ≥1 visits) during the follow up period was compared using chi-squared tests. In the pre/post-index analyses, the numbers of all-cause HCRU encounters were compared before and after epilepsy index date using repeated measures *t*-tests. Prevalence of HCRU during the baseline and follow up periods were compared using chi-squared tests. All analyses were performed using SAS version v9.4 (Cary, NC).

## 3 Results

A total of 126 individuals were identified with a RTT ICD-10 code. Of these, 28 individuals were excluded based on the absence of confirmed RTT in the clinical progress note. A total of 98 individuals met study eligibility criteria and were included in the final analyses (Figure 1). There were 71 (72%) RTT individuals with epilepsy, of which 33 (46.5%) had documentation of severe epilepsy (Figure 1). Of the 71 individuals with epilepsy, 29 (40.8%) had focal epilepsy, 23 (32.4%) had generalized epilepsy, and 19 (26.8%) were unspecified with regards to focal or generalized epilepsy. Methods of epilepsy identification are shown in Supplementary Table 2, where epilepsy was identified using encounter diagnosis (68.4%), clinical notes (52.0%), and epilepsy medication (28.6%). Methods of identification of individuals with severe epilepsy are shown in Supplementary Figure 1 and include clinical progress notes alone (3 of 33), ICD-10 codes alone (19 of 33), and both clinical progress notes and ICD-10 codes (11 of 33). Of the 98 individuals with RTT, majority were female (*n* = 91, 92.9%), and most were of white race (*n* = 60, 61.2%); 30 (30.6%) did not have race documented (Table 1). Prevalence of select comorbid conditions included dysphagia (40.8%), constipation (37.8%), gastroesophageal reflux disorder [GERD] (37.8%), scoliosis (36.7%), vomiting (30.6%), sleep apnea (30.6%), cardiac arrhythmias (21.4%), and QT prolongation (12.2%) (Table 1).

**Table 1:** Baseline patient demographic and clinical characteristics among total RTT (*n* = 98).

**Patient demographic and clinical characteristics**	**Total RTT cohort (*n* = 98)**
**Sex**, ***n*** **(%)**	
Male	7 (7.1%)
Female	91 (92.9%)
Age at initial epilepsy diagnosis, mean (SD)^*^	8.6 (9.2)
**Race**, ***n*** **(%)**	
Asian	1 (1.1%)
Black	6 (6.8%)
Multiple races	1 (1.0%)
Unknown	30 (30.6%)
White	60 (61.2%)
**Epilepsy and seizures**, ***n*** **(%)**	
Epilepsy	71 (72.4%)
Focal epilepsy	29 (29.6%)
Generalized epilepsy	23 (23.5%)
Epilepsy, unknown whether focal or generalized	19 (19.4%)
Severe epilepsy	33 (33.7%)
**Behavior and psychiatric comorbidities**, ***n*** **(%)**	
Anxiety	16 (16.3%)
Behavioral disorders and disturbances	16 (16.3%)
**Cardiac**, ***n*** **(%)**	
Cardiac arrhythmias	21 (21.4%)
QT prolongation	12 (12.2%)
**GI and nutrition comorbidities**, ***n*** **(%)**	
Constipation	39 (37.8%)
Dysphagia	40 (40.8%)
Gastrostomy	27 (27.6%)
GERD	37 (37.8%)
Nutritional deficiency	33 (33.7%)
Vomiting	30 (30.6%)
**Musculoskeletal**, ***n*** **(%)**	
Scoliosis	36 (36.7%)
Kyphosis and other spinal deformities	21 (21.4%)
Neurologic^**^, ***n*** **(%)**	
Movement disorders	15 (15.3%)
Weakness or paralysis	32 (32.7%)
**Respiratory**, ***n*** **(%)**	
Asthma	11 (11.2%)
Sleep apnea	30 (30.6%)

^*^Age among RTT individuals with epilepsy cases; ^**^other than epilepsy or seizures; GI, gastrointestinal; GERD, gastroesophageal reflux disorder; SD, standard deviation.

There were 59 RTT individuals with epilepsy who had 12 months of post-index follow-up of clinical history following the index epilepsy diagnosis. Table 2 displays the prevalence of providers visited and medications prescribed during the follow-up period among the 59 RTT individuals. The most commonly prescribed AEDs were levetiracetam (45.8%) followed by clonazepam (37.3%), oxcarbazepine (30.5%) (Table 2).

**Table 2:** Anti-epileptic drugs during 12-month follow up period (*n* = 59).

**Epilepsy medications, *n* (%)**	***N* = 59**
Levetiracetam	27 (45.8%)
Clonazepam	22 (37.3%)
Oxcarbazepine	18 (30.5%)
Lamotrigine	15 (25.4%)
Valproate/valproic acid	15 (25.4%)
Topiramate	10 (16.9%)
Lacosamide	7 (11.9%)
Parampanel	3 (5.1%)
Carbamazepine	2 (3.4%)
Fosphenytoin	1 (1.7%)
Phenytoin	1 (1.7%)

**Figure 2:**
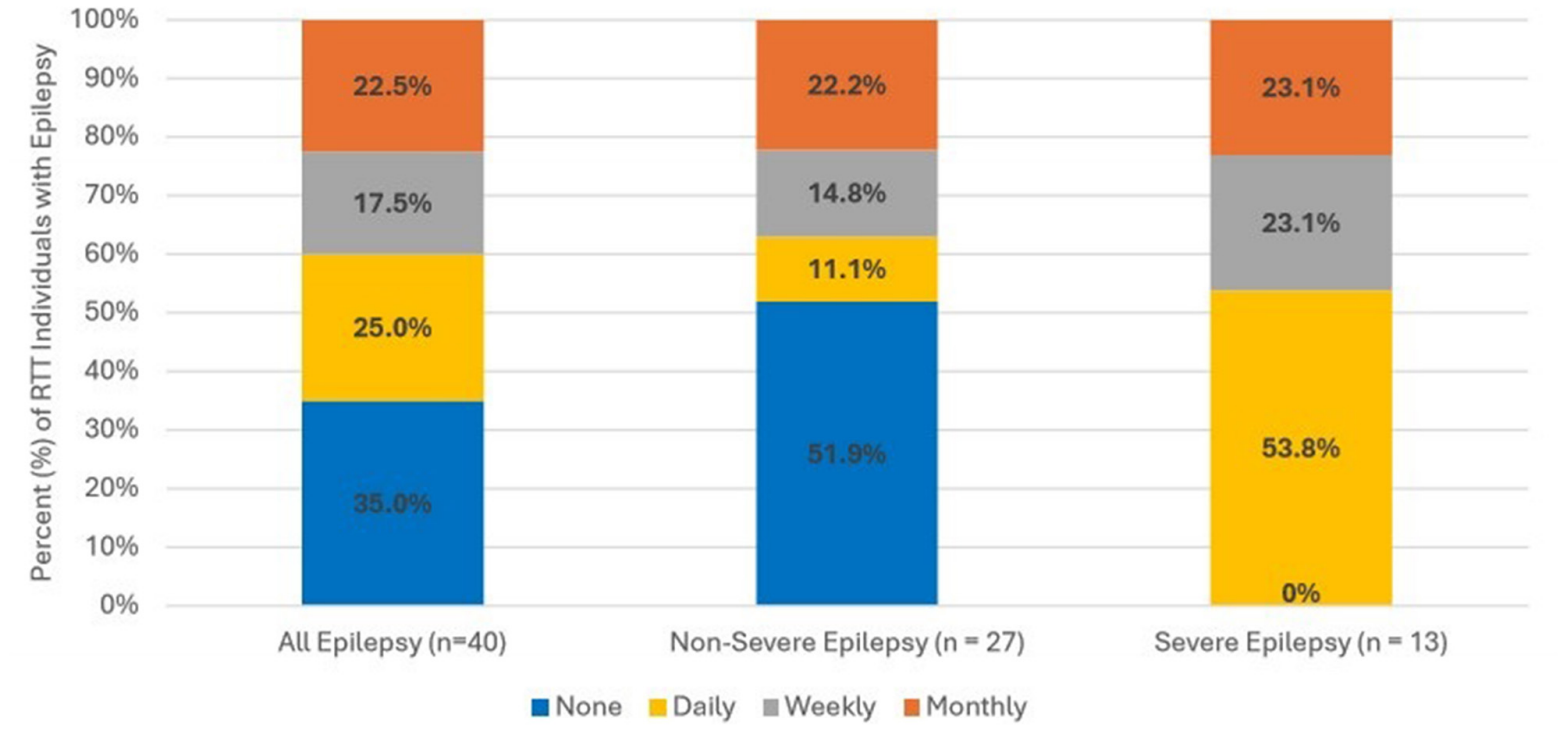
Frequency of seizures among RTT individuals with epilepsy. Seizure frequency information was available from clinical progress notes for 40 RTT individuals. Frequency was assessed with or without medication.

Figure 2 displays seizure frequency among overall epilepsy RTT individuals. There were 40 RTT individuals with documentation from clinical notes categorizing seizure frequency. Overall, one quarter (25.0%) of RTT individuals with epilepsy experienced daily seizures. Frequent seizures were more common among individuals with severe epilepsy relative to those with non-severe epilepsy. All individuals with severe epilepsy experienced seizures daily (53.8%), weekly (23.1%), or monthly (23.1%); while over half (51.9%) of individuals with non-severe epilepsy experienced no seizures during 12 months post-index. Among RTT individuals with non-severe epilepsy, 11.1% experienced daily seizures, 14.8% experienced weekly seizures, and 22.2% had monthly seizures.

**Figure 3:**
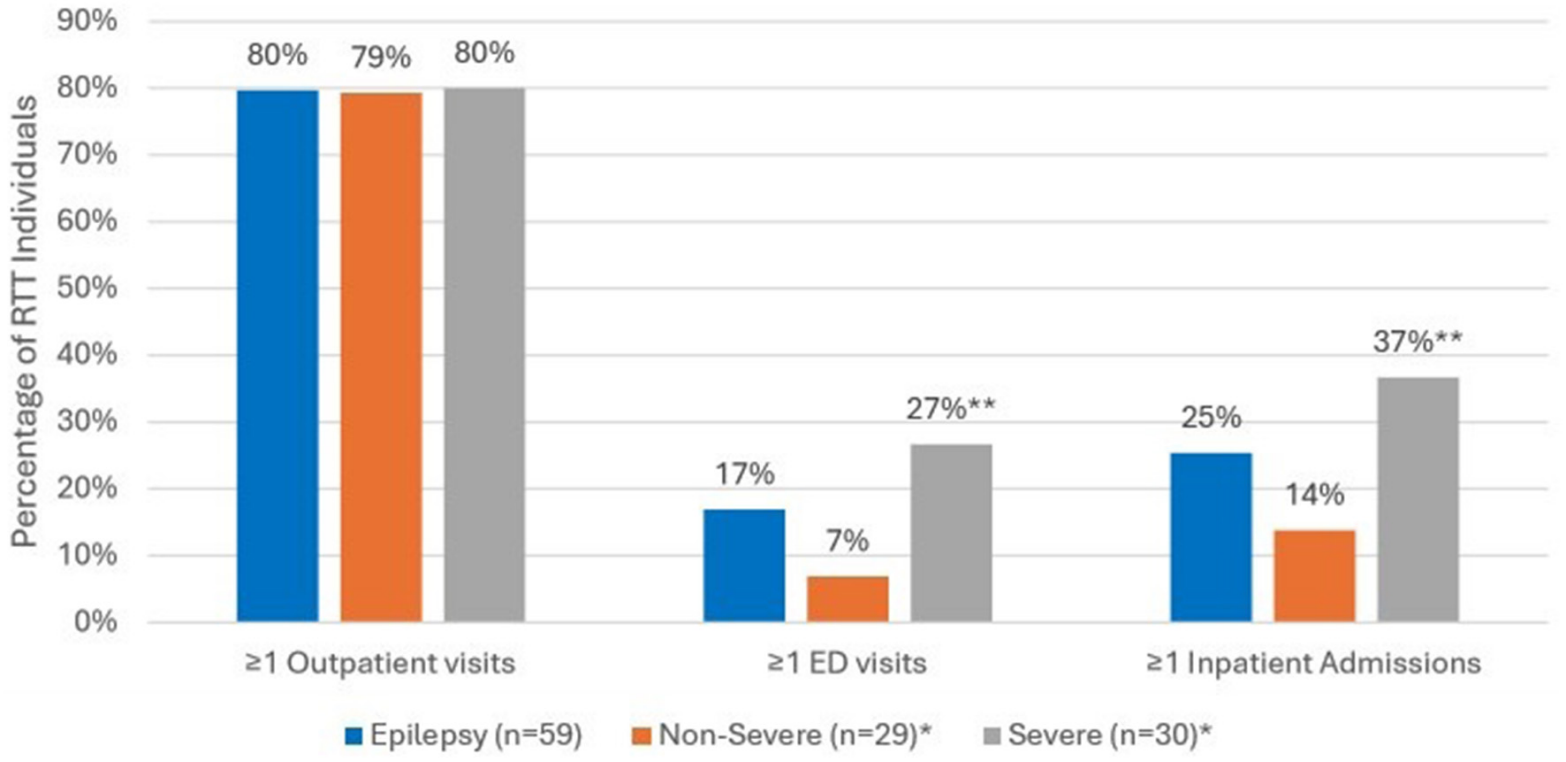
All-cause HCRU among RTT individuals with epilepsy (*n* = 59) during 12-month follow-up period. *Sub-categories of epilepsy group; **p < 0.05 demonstrating statistical significance between non-severe vs. severe group.

**Figure 4:**
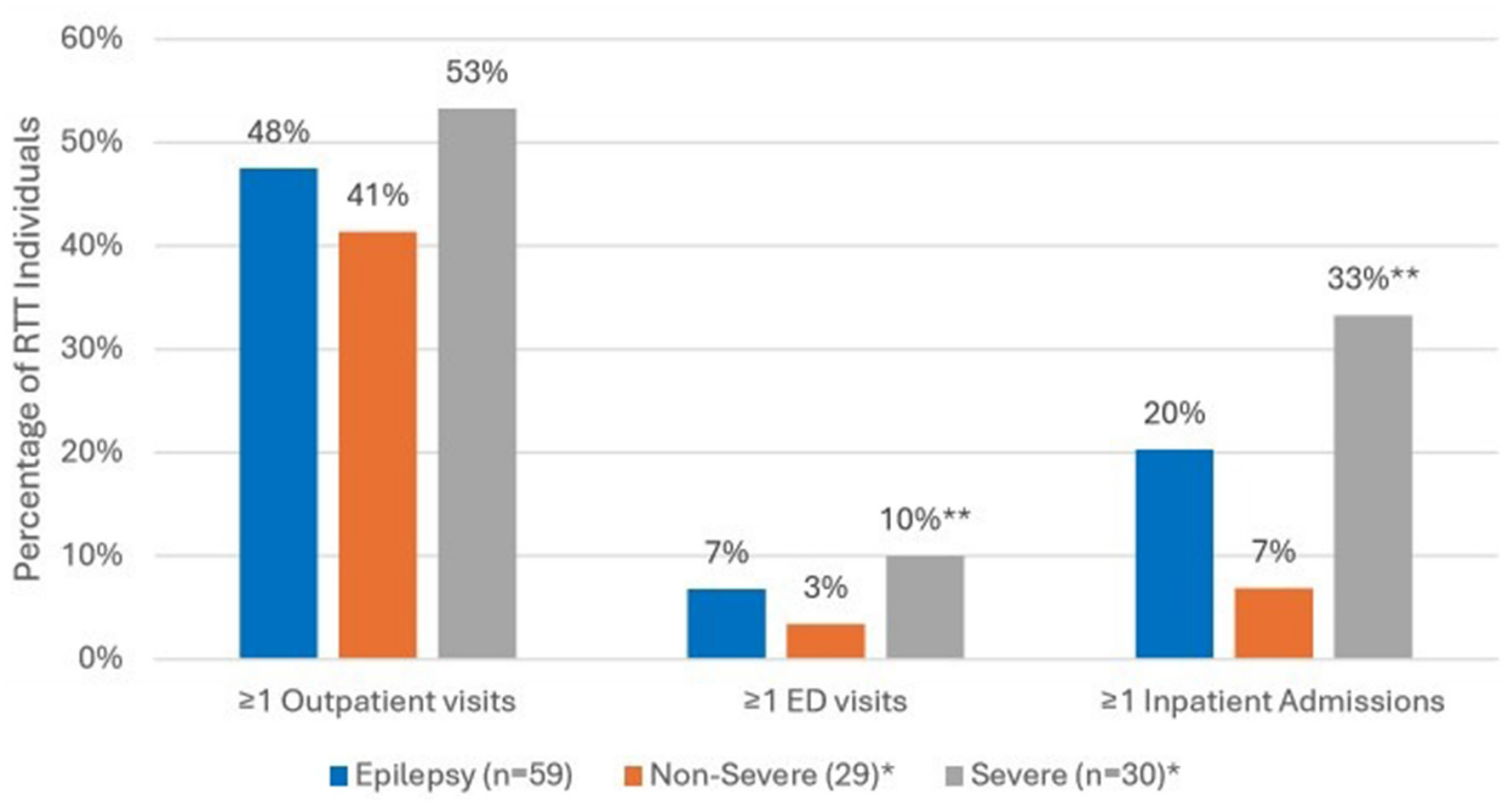
Epilepsy-specific HCRU in the 12-month follow up period among RTT individuals with Epilepsy (*n* = 59). *Sub-categories of epilepsy group; **p < 0.05 demonstrating statistical significance between non-severe vs. severe group; ED, emergency department.

Comparing all-cause HCRU during the 12-month follow up period (Figure 3), the prevalence of ED visits was significantly higher among those with severe epilepsy vs. those with non-severe epilepsy (27% vs. 7%, *p* < 0.05). Inpatient admissions were also significantly more prevalent among those with severe epilepsy vs. non-severe epilepsy (37% vs. 14%, *p* < 0.05). There were no significant differences in the prevalence of outpatient visits across the two groups. In the year following diagnosis, epilepsy-specific inpatient admissions were documented for 12 (20%) and ED visits for 4 (7%) of the 59 individuals with epilepsy (Figure 4). Compared to individuals with non-severe epilepsy, individuals with severe epilepsy were significantly more likely to have an epilepsy-specific inpatient admissions (33% vs. 7%; *p* < 0.05). Though not statistically significant (NS), individuals with severe epilepsy were also more likely to have ≥1 ED visits (10% vs. 3%, NS).

**Figure 5:**
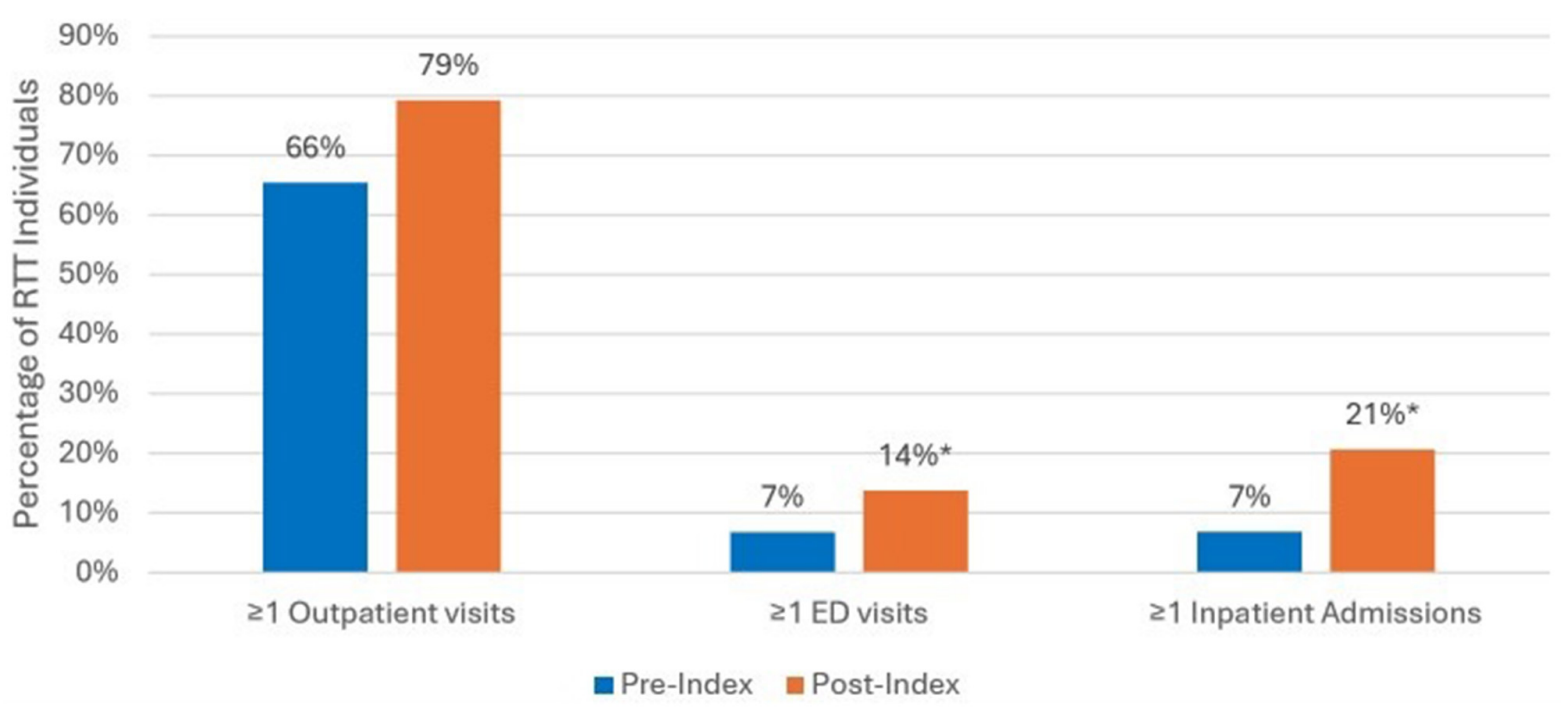
All-cause HCRU among RTT individuals with epilepsy for those with 12 months pre/post-index analysis (*n* = 29). *p < 0.05 demonstrating statistical significance between pre-index vs. post-index.

Figure 5 displays the results of the 12-month pre/post-index analyses comparing all-cause HCRU before and after epilepsy diagnosis. Among the 71 individuals with documented epilepsy, a total of 29 (41%) had clinical history during the 12 months pre-index and 12 months post-index. There was an increase of inpatient admissions post-epilepsy diagnosis during the 12 months post-index vs. 7% during the 12 months pre-index (*p* < 0.05). The trend was similar for outpatient and ED visits, but not statistically significant; during the baseline 12 month prior to initial diagnosis compared to the follow up period, higher percentages of RTT individuals had ≥1 ED visits during the 12 months post-index compared to pre-index, 14% vs. 7% (NS), respectively; and higher outpatient visits during post-index compared to pre-index, 79% vs. 66 % (NS), respectively.

## 4 Discussion

To date, there are no published studies examining epilepsy outcomes and related HRCU in individuals with RTT using a combination of structured EHR data and unstructured data abstracted from clinical progress notes from a RTT COE. In our study population, epilepsy was common (72%), with close to half (46%) experiencing prolonged, cyanotic, or intractable seizures. In this study, the rate of daily seizures (25%) among individuals with epilepsy was higher compared to previously reported studies of 11% (8). Daily seizures are associated with increased caregiver burden and lower emotional quality of life (15). While the elevated frequency of seizures among the study population may be a result of selection bias due to a more profoundly affected RTT population that seeks care at a COE or a limitation of the available seizure frequency data, seizure frequency reported by parents of children with epilepsy tend to underestimate actual seizure frequency (16).

This study highlights the risk and burden to patients and families associated with epilepsy, especially those with refractory, cyanotic, or prolonged seizures. In this study, individuals with these types of severe seizures were 3.9 times more likely to visit an ED and 2.5 times more likely to be admitted to a hospital in the year post initial diagnosis. Clinically, the consequences of refractory seizures include increased risk for neuropsychiatric complications (e.g., ADHD, depression) and decreased life expectancy (17). Individuals who experience prolonged seizures and/or status epilepticus are increased risk for pneumonitis and aspiration pneumonia, respiratory failure, neurological deficits (hemiparesis, cognitive decline, dysphasia), fractures, decreased growth, and increased mortality (3, 6, 18–20). It is not surprising that individuals with RTT who also experience these more serious forms of epilepsy are more likely to require emergent and inpatient care. However, seizures appear to contribute to caregiver burden even in non-severe forms (21).

The most common causes of mortality in individuals with RTT are respiratory-related including lower respiratory tract infection, aspiration/asphyxiation, and respiratory failure (22). There is a strong link between aspiration and subsequent respiratory failure in individuals with RTT (23). Individuals with RTT have multiple comorbidities that put them at risk for aspiration and subsequent respiratory complications including dysphagia, GERD, scoliosis as well as epilepsy (23). Gastrointestinal comorbidities, also common in this RTT cohort, can also be a source of inpatient admissions and ED visits (24, 25). The increased healthcare utilization in individuals with RTT and epilepsy found in this study is likely not an isolated phenomenon but rather the result of interactions between multiple comorbid conditions.

While the diagnosis of RTT is made clinically, there are strong associations with genetic mutations and clinical presentation including seizure onset and severity. Individuals with CDKL5 and FOXG1 mutations appear to have earlier onset of seizures and those with large MECP2 gene deletions have later onset (11, 26). Individuals without identifiable genetic mutations appear to be at lower risk for epilepsy (11). While beyond the scope of this study, future research is warranted to explore the interaction between RTT genetic variant, epilepsy severity, healthcare utilization and clinical burden. While AEDs are effective therapy, either as monotherapy or in combination, for many individuals who have seizures in RTT, 20–40% experience drug-resistant epilepsy (27). While almost all the most common AEDs for long-term seizure control reported in our study (levetiracetam, oxcarbazepine, lamotrigine, valproic acid, and topiramate) are recommended in one or more clinical guidelines for epilepsy in RTT, with the exception of oxcarbazepine, specific recommendations regarding first-line agents or care pathways are lacking (28). Due to the non-localized nature of epilepsy in RTT, unlike individuals with other types epilepsy (i.e., focal onset epilepsy), surgical treatments are generally not effective for drug-resistant epilepsy in RTT, though corpus callosotomy has been used successfully in select cases (27, 29, 30).

The additional burden placed upon families with RTT who also have seizures, especially among those with refractory and/or frequent, prolonged seizures, is substantial and can result in caregiver anxiety, depression, and decreased quality of life (31, 32). Seizures are the second most reported concern by caregivers in RTT, second only to lack of effective communication. Parental concerns related to seizures tend to be minor among children under the age of 5, grow until they peak at 15–20 years of age, and slowly decline after age 20 (33). Increasing seizure frequency in RTT is directly associated with increased caregiver burden (34). AED treatment can also add burden to patients and families. Use of multiple AEDs is associated with increased caregiver burden (32), and many individuals experience significant side effects including behavioral disturbances, anorexia, nephrolithiasis, and sedation (3). Interventions such as music therapy, reported to reduce caregiver stress and reduce seizure frequency, can be an important component of a RTT treatment plan (35). Thus, to control or stabilize seizures, especially refractory seizures in RTT, have the potential to reduce unwanted healthcare utilization and profoundly improve the quality of life for individuals with RTT and their families.

There are several limitations to address. This study was performed based upon data from a single health system whose coding and documentation practices may differ from other health providers. This study was also limited to clinical encounters and documentation related to care received at VUMC and may not represent the full healthcare experience. In addition, the population of VUMC individuals with RTT is relatively small and has substantial variability in available data, limiting the ability of this study to detect significant differences between cases and comparators. VUMC is a RTT COE and their population of RTT individuals may differ from other health systems. The RTT population seeking care at a RTT COE may represent a cohort of individuals with more severe RTT with more disabling disease and more symptomatic epilepsy. As a result, the results presented in the study may not represent the experience of individuals with RTT cared for at other clinical settings. Additionally, the sample sizes of the cohorts and sub cohorts in this study are small, and as a result, limits the generalizability of our findings. For example, the results of the pre- post-longitudinal analysis, limited to a subpopulation of 29 individuals, while suggestive of increased utilization associated with incident epilepsy, should be interpreted with caution and validated with a larger sample size. High-quality, rich clinical datasets for rare conditions such as RTT can be associated with sample limitations. While the authors sought to maximize the value of the available data using multiple study designs, analyses of larger populations of individuals with RTT are warranted.

RTT is also a heterogeneous condition with typical and atypical variants, different genetic mutations, and numerous comorbidities which can impact seizure activity and healthcare utilization. Exploration of these clinical and genetic features is beyond the scope of this study and deserves further analysis (6, 23). With regards to the accuracy of parental report of seizure activity, manifestations of RTT such as breath holding, staring, and unusual movements can be mistaken for seizures if not appropriately correlated with EEG-video monitoring (9). Conversely, parents of children with epilepsy commonly under-report seizure activity, both when parents are able and not able to directly observe their child (16). Confirmation of seizure frequency with EEG-video monitoring, however, was beyond the scope of this initiative.

There are strengths to mention such as real-world evidence studies of individuals with RTT are limited and this study of epilepsy in individuals with RTT is the first to assess associated HCRU at a RTT COE. The combination of structured and unstructured data provides greater understanding of seizure frequency and severity that structured data alone.

## 5 Conclusion

Among the RTT COE data, over 70% of individuals with RTT had comorbid epilepsy and 25% experienced daily seizures. Individuals with RTT and refractory or prolonged seizures were more likely to experience inpatient admissions and ED visits. It is important to recognize that individuals with RTT have high concomitant rates of epilepsy, and to alleviate additional burden among caregivers and patients, the goal to stabilize the frequency of seizures is essential; especially for those with severe epilepsy.

## Data Availability

The datasets presented in this article are not readily available because the data that support the findings of this study are available from Nashville Biosciences but restrictions apply to the availability of these data, which were used under license for the current study, and so are not publicly available. Data are however available with permission from Acadia Pharmaceuticals. Requests to access the datasets should be directed to Nazia Rashid, nrashid@acadia-pharm.com.

## References

[1] Neul JL Kaufmann WE Glaze DG Christodoulou J Clarke AJ Bahi-Buisson N. Rett syndrome: revised diagnostic criteria and nomenclature. Ann Neurol. (2010) 68:944–50. 10.1002/ana.22124 21154482 PMC3058521

[2] Petriti U Dudman DC Scosyrev E Lopez-Leon S. Global prevalence of Rett syndrome: systematic review and meta-analysis. Syst Rev. (2023) 12:5. 10.1186/s13643-023-02169-6 36642718 PMC9841621

[3] Tarquinio DC Hou W Neul JL Lane JB Barnes KB O'Leary HM. Age of diagnosis in Rett syndrome: patterns of recognition among diagnosticians and risk factors for late diagnosis. Pediatr Neurol. (2015) 52:585–91.e2. 10.1016/j.pediatrneurol.2015.02.007 25801175 PMC4442062

[4] Einspieler C Marschik PB. Regression in Rett syndrome: developmental pathways to its onset. Neurosci Biobehav Rev. (2019) 98:320–32. 10.1016/j.neubiorev.2019.01.028 30832924

[5] Pini G Bigoni S Congiu L Romanelli AM Romanelli AM Di Marco P. Rett syndrome: a wide clinical and autonomic picture. Orphanet J Rare Dis. (2016) 11:132. 10.1186/s13023-016-0499-7 27682832 PMC5041273

[6] Fu C Armstrong D Marsh E Lieberman D Motil K Witt R. Multisystem comorbidities in classic Rett syndrome: a scoping review. BMJ Paediatr Open. (2020) 4:e000731. 10.1136/bmjpo-2020-000731 33024833 PMC7509967

[7] Tarquinio DC Hou W Berg A Kaufmann WE Lane JB Skinner SA. Longitudinal course of epilepsy in Rett syndrome and related disorders. Brain. (2017) 140:306–18. 10.1093/brain/aww302 28007990 PMC5278305

[8] Glaze DG Percy AK Skinner S Motil KJ Neul JL Barrish JO. Epilepsy and the natural history of Rett syndrome. Neurology. (2010) 74:909–12. 10.1212/WNL.0b013e3181d6b852 20231667 PMC2836870

[9] Bricker K Vaughn BV. Rett syndrome: a review of clinical manifestations and therapeutic approaches. Front Sleep. (2024) 3:1373489. 10.3389/frsle.2024.1373489 PMC12713886 41424498

[10] Jian L Nagarajan L de Klerk N Ravine D Christodoulou J Leonard H. Seizures in Rett syndrome: an overview from a one-year calendar study. Eur J Paediatr Neurol. (2007) 11:310–7. 10.1016/j.ejpn.2007.02.008 17433737 PMC3013620

[11] Operto FF Mazza R Pastorino GMG Verrotti A Coppola G. Epilepsy and genetic in Rett syndrome: a review. Brain Behav. (2019) 9:e01250. 10.1002/brb3.1250 30929312 PMC6520293

[12] Cardoza B Clarke A Wilcox J Gibbon F Smith PE Archer H. Epilepsy in Rett syndrome: association between phenotype and genotype, and implications for practice. Seizure. (2011) 20:646–9. 10.1016/j.seizure.2011.06.010 21764336

[13] Spagnoli C Fusco C Pisani F. Rett syndrome spectrum in monogenic developmental-epileptic encephalopathies and epilepsies: a review. Genes. (2021) 12:1157. 10.3390/genes12081157 34440332 PMC8394997

[14] Bahi-Buisson N Guellec I Nabbout R Guet A Nguyen G Dulac O. Parental view of epilepsy in Rett syndrome. Brain Dev. (2008) 30:126–30. 10.1016/j.braindev.2007.07.002 17707604

[15] Abd Elmoaty A Salem N. Burden and quality of life among caregivers to children with epilepsy. J Nurs Res. (2019) 7:817–23. 10.12691/ajnr-7-5-15

[16] Akman CI Montenegro MA Jacob S Eck K Chiriboga C Gilliam F. Seizure frequency in children with epilepsy: factors influencing accuracy and parental awareness. Seizure. (2009) 18:524–9. 10.1016/j.seizure.2009.05.009 19592270

[17] Laxer KD Trinka E Hirsch LJ Cendes F Langfitt J Delanty N. The consequences of refractory epilepsy and its treatment. Epilepsy Behav. (2014) 37:59–70. 10.1016/j.yebeh.2014.05.031 24980390

[18] Tortuyaux R Wallet F Derambure P Nseir S. Bacterial aspiration pneumonia in generalized convulsive status epilepticus: incidence, associated factors and outcome. J Clin Med. (2022) 11:6673. 10.3390/jcm11226673 36431150 PMC9695142

[19] Hawkes MA Hocker SE. Systemic complications following status epilepticus. Curr Neurol Neurosci Rep. (2018) 18:7. 10.1007/s11910-018-0815-9 29417304

[20] Lu M Faure M Bergamasco A Spalding W Benitez A Moride Y. Epidemiology of status epilepticus in the United States: a systematic review. Epilepsy Behav. (2020) 112:107459. 10.1016/j.yebeh.2020.107459 33181886

[21] Kaufmann WE Percy AK Neul JL Downs J Leonard H Nues P. Burden of illness in Rett syndrome: initial evaluation of a disorder-specific caregiver survey. Orphanet J Rare Dis. (2024) 19:296. 10.1186/s13023-024-03313-8 39138481 PMC11323357

[22] Anderson A Wong K Jacoby P Downs J Leonard H. Twenty years of surveillance in Rett syndrome: what does this tell us? Orphanet J Rare Dis. (2014) 9:87. 10.1186/1750-1172-9-87 24942262 PMC4078387

[23] Rashid N Darer JD Ruetsch C Yang X. Aspiration, respiratory complications, and associated healthcare resource utilization among individuals with Rett syndrome. Orphanet J Rare Dis. (2025) 20:232. 10.1186/s13023-025-03757-6 40375331 PMC12082938

[24] Darer J May D Rashid N Kyle S Yang X Ruetsch C. Gastrointestinal symptoms and healthcare utilization among individuals with Rett syndrome at a center of excellence. Fut Rare Dis. (2025) 5:2474364. 10.1080/23995270.2025.2474364

[25] May DM Neul J Piña-Garza JE Kponee-Shovein K Satija A Mahendran M. Gastrointestinal manifestations in pediatric and adult patients with Rett syndrome: an analysis of US claims and physician survey data. J Comp Eff Res. (2024) 13:e230054. 10.57264/cer-2023-0054 37971297 PMC10842289

[26] Pintaudi M Calevo MG Vignoli A Parodi E Aiello F Baglietto MG. Epilepsy in Rett syndrome: clinical and genetic features. Epilepsy Behav. (2010) 19:296–300. 10.1016/j.yebeh.2010.06.051 20728410

[27] Krajnc N. Management of epilepsy in patients with Rett syndrome: perspectives and considerations. Ther Clin Risk Manag. (2015) 11:925–32. 10.2147/TCRM.S55896 26089674 PMC4468994

[28] Amin S Ruban-Fell B Newell I Evans J Vyas K Nortvedt C. Treatment guidelines for rare, early-onset conditions associated with epileptic seizures: a literature review on Rett syndrome and tuberous sclerosis complex. Orphanet J Rare Dis. (2024) 19:89. 10.1186/s13023-023-02994-x 38409029 PMC10895812

[29] Datta A Tamber M. Case report: successful complete open corpus callosotomy for refractory epilepsy in Rett syndrome. Brain Dev Case Rep. (2024) 2:100032. 10.1016/j.bdcasr.2024.100032

[30] Ueda K Sood S Asano E Kumar A Luat AF. Elimination of medically intractable epileptic drop attacks following endoscopic total corpus callosotomy in Rett syndrome. Childs Nerv Syst. (2017) 33:1883–7. 10.1007/s00381-017-3567-y 28815309 PMC9728060

[31] Pokharel R Poudel P Lama S Thapa K Sigdel R Shrestha E. Burden and its predictors among caregivers of patient with epilepsy. J Epilepsy Res. (2020) 10:24–30. 10.14581/jer.20005 32983952 PMC7494882

[32] Karakis I Cole AJ Montouris GD San Luciano M Meador KJ Piperidou C. Caregiver burden in epilepsy: determinants and impact. Epilepsy Res Treat. (2014) 2024:808421. 10.1155/2014/808421 24808956 PMC3997889

[33] Neul JL Benke TA Marsh ED Suter B Silveira L Fu C. Top caregiver concerns in Rett syndrome and related disorders: data from the US natural history study. J Neurodevelop Disord. (2023) 15:33. 10.1186/s11689-023-09502-z 37833681 PMC10571464

[34] Killian JT Jr Lane JB Lee HS Pelham JH Skinner SA Kaufmann WE. Caretaker quality of life in Rett syndrome: disorder features and psychological predictors. Pediatr Neurol. (2016) 8:67–74. 10.1016/j.pediatrneurol.2015.12.021 26995066 PMC4899118

[35] Chou MY Chang NW Chen C Lee WT Hsin YJ Siu KK. The effectiveness of music therapy for individuals with Rett syndrome and their families. J Formos Med Assoc. (2019) 118:1633–43. 10.1016/j.jfma.2019.01.001 30670340

