# Supplementary material for: Prevalence, severity, frequency and healthcare resource use of epilepsy among individuals with Rett Syndrome: analysis of data from a Rett Center of Excellence
Source: Front Neurol. 2025 Oct 10;16:1634105. doi: 10.3389/fneur.2025.1634105 (PMC12549289; doi:10.3389/fneur.2025.1634105)

Supplementary Material

# Supplementary Tables

Supplemental Table 1. Comorbidities and Associated ICD-9 and ICD-10 Codes

| **Clinical Domain** | **Diagnoses** | **ICD-9 or ICD-10 Code** |
| --- | --- | --- |
| Epilepsy | Epilepsy and recurrent seizures | 345.XX, G40.XX |
| Severe epilepsy | Intractable epilepsy | 345.X1, G40.3X, G40A1X, G40B1X, G40.C1X, G40.41X, G40.8X3, G40.8X4, G40.91X |
| Severe epilepsy | Status epilepticus | G40.XX1 |
| Behavior and psychiatric | Anxiety | 300.0X, F06.4, F41.X |
| Behavior and psychiatric | Behavioral disorders (e.g., aggression) | 294.11, 312.X, F90.X, F91.X, F94.X, F95.X, F98.XX, R45.1, R45.4-R45.6, R45.83 |
| Cardiac | Cardiac arrhythmias | I49.9, 427.9, 427.89 |
| Cardiac | QT Prolongation | I45.81, 426.82 |
| GI and Nutrition | Constipation | K59.00, K59.01, K59.04, K59.09, 564.00, 564.01, 564.09 |
| GI and Nutrition | Dysphagia | R13.1X, 787.2X |
| GI and Nutrition | Gastrostomy | 536.4X, Z93.1, K94.2X |
| GI and Nutrition | GERD | 530.81, 530.11, K21.XX |
| GI and Nutrition | Vomiting | 787.01, 787.03, R11.1X, R11.2 |
| GI and Nutrition | Hematemesis | 578.0, K92.0 |
| GI and Nutrition | Nutritional deficiency and failure to thrive | 26X.XX, 783.22, 783.41, 783.7, E40, E41, E42, E43, E44, E45, E46, E63.X, R62.51, R62.7 |
| Musculoskeletal | Kyphosis and other spinal deformities | R29.3, M43.9, M40.05, M40.204, Q67.5, M40.14, 754.2, M40.57, M40.209, Q76.414, 737.29, 781.92 |
| Musculoskeletal | Scoliosis | 737.3X, 737.43, M41.XX |
| Neurologic | Movement disorders | 781, G25.5, G26, G25.9, G47.61, R25.8 |
| Neurologic | Weakness or paralysis | M62.81, 728.87, G82.50, 343.2, P94.2, 342.11, 342.12, 334.1 |
| Respiratory | Asthma | 493.XX, J45.XX, 518.3 |
| Respiratory | Sleep apnea | 327.2X, 780.51, 780.53, 780.57, G47.3X |

Supplemental Table 2. Methods of identification of epilepsy among individuals with RTT

| Methods of Identification, n (%) | Total RTT Cohort  N=98 |
| --- | --- |
| Encounter diagnosis of epilepsy | 67 (68.4%) |
| Clinical note documenting epilepsy | 51 (52.0%) |
| Epilepsy medication | 28 (28.6%) |
| At least one indication of epilepsy from above | 71 (72.4%) |

Supplemental Figure 1. Identification of individuals with Severe Epilepsy


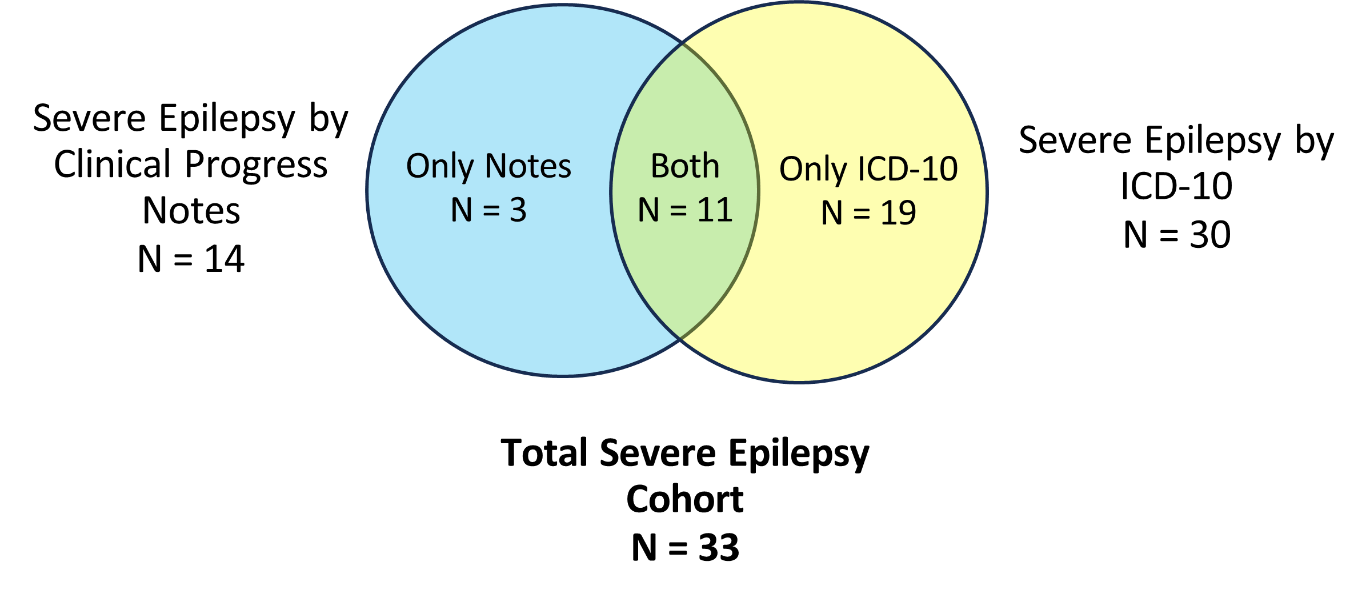

Supplement: Supplementary file 1 [file Table_1.docx]
